# Medical Opinion and Movement

**Published:** 1907-05-18

**Authors:** 


### MEDICAL OPINION AND MOVEMENT.

AT a meeting on May 2 at the Royal United Service Institute, attended by a large number of medical officers of the Army and Navy, a new society was formed, which we hope may be productive of much good. The society is to be called "The United Services Medical Society," and will consist of medical officers of the navy, British and Indian armies, and the Auxiliary and Colonial forces. The objects of the society, as stated by the Chairman of the meeting, Inspector-General H. M. Ellis, Director-General of the Medical Department of the Navy, are: (1) The furtherance of sciences bearing upon the preservation of the health of the forces; (2) the study of diseases and injuries incidental to the life of sailors and soldiers and the treatment of the same; (3) the study of organisations, methods and apparatus for the amelioration of the condition of the wounded in war; (4) all matters coming within the scope of the technical duties of naval and military medical officers. The meetings will be held monthly in the Royal Army Medical College. The society will be managed by a council, and Inspector-General Ellis was elected President for the ensuing year.

As a result of a careful investigation into the bacteriology of pertussis, Professor Albrecht, of Vienna, has formed the somewhat surprising conclusion that the bacillus pertussis of Eppendorf is identical with the bacillus influenzæ. He has found the bacillus pertussis present in 200 cases of fatal pneumonia following whooping-cough in children, and in the sputum of 70 children actually suffering from whooping-cough. He has also isolated the same bacillus from abscesses complicating pertussis and from cases of bronchitis and pneumonia after measles, diphtheria, and influenza. In no case was any difference found between the organisms obtained from usual influenza and from these cases, and he is of opinion that they cannot be distinguished morphologically or biologically. Rabbits immunised with this bacillus showed a high agglutination index. Intravenous injections of broth cultures frequently gave rise to endocarditis of the mitral valves and degeneration of the liver and heart-muscles, and large quantities of the bacillus influenza were obtained from these parts. These facts would explain the severe cardiac complications which so often accompany comparatively mild attacks of influenza. It will be highly interesting to see what light further bacteriological research may throw upon the question of the identity of these two micro-organisms, but in the meantime it is difficult to reconcile our clinical knowledge of the two diseases with a common microbic origin.

THE Liverpool School of Tropical Medicine has not only distinguished itself by the way in which it equips medical men with the necessary knowledge to practise their profession in the tropical parts of the British dominions, but it also forms one of the centres of keenest activity in carrying out scientific research by organising expeditions to combat disease. The latest expedition has been despatched to Central Africa to study sleeping-sickness, and advise on means by which it can be prevented from spreading to regions not yet infected. Dr. Allan Kinghorn and Mr. R. Eustace Montgomery are taking part in this expedition, and they will visit British East Africa, British Central Africa, Rhodesia, the Portuguese territories, and the Congo Free State. The Cape Government will give them free transport over their lines, and every assistance will be offered them by the Governments and corporate bodies of those parts. A farewell banquet was given to the expedition by Sir Alfred Jones, President of the School, and messages were read from the Earl of Derby, Lord Elgin, and Mr. J. Chamberlain. In the course of his speech Sir Alfred Jones said that they had sent out 22 expeditions from the School at a cost of £80,000. They were now organising another expedition to study blackwater fever, and this would leave next August.

AN accurate knowledge of the anatomy of the lymphatic system, of the course of the lymphatic vessels, and of their connections with the gland groups in the different parts of the body, is of the utmost importance to a proper understanding of the spread of microbic infections and of malignant disease. Mr. W. S. Handley has done excellent work in this field by demonstrating the lymphatic connections of the breast with adjacent parts of the body, and he was able in consequence to indicate clearly the lines on which excision of the breast for carcinoma should be carried out in order to secure a thorough removal of the disease. Recently Messrs. J. Kay Jamieson and J. F. Dobson, of the Leeds University, have worked at the anatomy of the lymphatic system in relation to the stomach and the cæcum and appendix. In regard to the stomach, they are led to the conclusion that the operation of radical gastrectomy for carcinoma, as designed by Cuneo and carried out by Hartmann, Moynihan, and Mayo, does not ensure complete removal of affected glands even of the "first relay." Unfortunately, these glandular groups are so complex and extensive that a radical operation in the sense originally intended seems altogether beyond the powers of the surgeon, except in cases of a very early diagnosis, before any lymphatic invasion has taken place. Their researches on the lymphatic connections of the cæcum also show that the usual operation of excision does not ensure removal of these connections, and they have devised a method of procedure which would accomplish this purpose. Although this operation is more extensive than the one hitherto adopted, the authors consider that it would not involve greater risk to the patient, as the surgeon would be better able to control the blood-supply of the part, and to recognise and avoid important structures which may be wounded and have frequently been wounded in the past.

